# Evaluation of an Internet-Based Monitoring System for Influenza-Like Illness in Sweden

**DOI:** 10.1371/journal.pone.0096740

**Published:** 2014-05-13

**Authors:** Moa Rehn, AnnaSara Carnahan, Hanna Merk, Sharon Kühlmann-Berenzon, Ilias Galanis, Annika Linde, Olof Nyrén

**Affiliations:** 1 Public Health Agency of Sweden (Previously Swedish Institute for Communicable Disease Control), Solna, Sweden; 2 European Programme for Intervention Epidemiology Training (EPIET), European Centre for Disease Prevention and Control (ECDC), Stockholm, Sweden; 3 Department of Medical Epidemiology and Biostatistics, Karolinska Institute, Solna, Sweden; Fondazione Bruno Kessler, Italy

## Abstract

To complement traditional influenza surveillance with data on disease occurrence not only among care-seeking individuals, the Swedish Institute for Communicable Disease Control (SMI) has tested an Internet-based monitoring system (IMS) with self-recruited volunteers submitting weekly on-line reports about their health in the preceding week, upon weekly reminders. We evaluated IMS acceptability and to which extent participants represented the Swedish population. We also studied the agreement of data on influenza-like illness (ILI) occurrence from IMS with data from a previously evaluated population-based system (PBS) with an actively recruited random sample of the population who spontaneously report disease onsets in real-time via telephone/Internet, and with traditional general practitioner based sentinel and virological influenza surveillance, in the 2011–2012 and 2012–2013 influenza seasons. We assessed acceptability by calculating the participation proportion in an invited IMS-sample and the weekly reporting proportion of enrolled self-recruited IMS participants. We compared distributions of socio-demographic indicators of self-recruited IMS participants to the general Swedish population using chi-square tests. Finally, we assessed the agreement of weekly incidence proportions (%) of ILI in IMS and PBS with cross-correlation analyses. Among 2,511 invited persons, 166 (6.6%) agreed to participate in the IMS. In each season, 2,552 and 2,486 self-recruited persons participated in the IMS respectively. The weekly reporting proportion among self-recruited participants decreased from 87% to 23% (2011–2012) and 82% to 45% (2012–2013). Women, highly educated, and middle-aged persons were overrepresented among self-recruited IMS participants (p<0.01). IMS (invited and self-recruited) and PBS weekly incidence proportions correlated strongest when no lags were applied (r = 0.71 and r = 0.69, p<0.05). This evaluation revealed socio-demographic misrepresentation and limited compliance among the self-recruited IMS participants. Yet, IMS offered a reasonable representation of the temporal ILI pattern in the community overall during the 2011–2012 and 2012–2013 influenza seasons and could be a simple tool for collecting community-based ILI data.

## Introduction

The societal consequences of influenza – whether it is seasonal or pandemic – include loss of production, exhaustion of health care resources, and excess mortality [1]. When monitoring the recurring epidemics valid incidence data in close-to-real-time can be of value. Traditionally, influenza surveillance is based on information from health care-based sources, including clinical and virological data [2]. However, influenza is often mild, does not always require health care and the proportion of ill people who see their general practitioner (GP) may be context dependent [3]. Therefore, traditional influenza surveillance may not generate valid representations of an epidemic in the community. To supplement traditional influenza surveillance systems with community-based information, the Swedish Institute for Communicable Disease Control (SMI) has tested two different prospective ways of collecting data on influenza-like illness (ILI) from the general population. Since 2007, a population-based system (PBS) uses cohorts established through annual random sampling of the target population. The cohort members provide event-driven, self-initiated reports via automated technologies as soon as a respiratory tract infection occurs. The PBS has been evaluated previously [4], [5]. Since 2011, an Internet-based monitoring system (IMS) with self-recruited participants provides reports of participants’ recent health status, which is collected upon a weekly reminder automatically dispatched via e-mail. The latter method was developed in the Netherlands in 2003 [6], [7]. SMI has compared representativeness and the obtained surveillance data in IMS and PBS during two seasons within the EPIWORK project, a European commission seventh frame work programme consortium aiming to build the foundation for an infrastructure to generate epidemic forecasts [8]. We regarded PBS as the best available and most rigorously evaluated method for community-based surveillance and therefore used it as the reference standard to IMS.

The self-selection involved in the recruitment of IMS participants has raised concerns about the validity of obtained ILI incidence data and its ability to reflect epidemics in the community. Reports from IMS in other European countries suggest that the self-recruited sample misrepresents its target population, but that ILI patterns correlate well with health care-based ILI data [6], [7], [9]–[11]. However, comparison of IMS with corresponding data generated from the more representative PBS cohort may provide better insights about the validity of disease occurrence data and the need, if any, for calibration of ILI estimates to correct for potential systematic errors. In this study, we first assessed the acceptability of IMS among randomly selected individuals who were invited to participate. Second, we assessed the representativeness of self-recruited IMS participants and compared it to that of the invited participants. Third, we compared IMS and PBS data in terms of ILI occurrence across the 2011–2012 and 2012–2013 influenza seasons (henceforth referred to as season 1 and season 2, respectively). We also related data from both systems to concurrently collected data from regular influenza surveillance: the laboratory reports from routine diagnostics and the GP based sentinel surveillance [12].

## Methods

### Ethics Statement

The IMS and PBS were reviewed and approved by the Stockholm regional research ethics review board (IMS: 2011/387-31/4, 2012/1445-32/4 and PBS: 2007/952-31, 2007/1599-32, 2008/1227-32, 2009/752-31, 2010/237-31/5, 2012/1444-32/5).

### The Internet-based Monitoring System (IMS)

IMS is a Swedish adaptation of the European-wide Influenzanet [13], [14]. During season 1, we recruited participants using press releases and resulting media attention, and by interpersonal communication through social media channels (henceforth referred to as self-recruited participants). Interested presumptive participants were directed to the project website. In season 2, we re-contacted participants from the previous season via e-mail with an invitation to participate again and recruited new participants as described above.

In season 2 we also investigated the possibility of implementing a population-based variant of IMS. We drew a random sample of 2,511 persons of the Swedish population aged 3 months through 95 years from the population register and invited them by post to participate in IMS (henceforth referred to as invited participants). The distribution of socio-demographic indicators of the random sample are available in Table S1.

Both self-recruited and invited participants (henceforth referred to as all IMS participants) initiated their participation by providing an e-mail address in a password-protected user account and by completing a background questionnaire on the project website. A user account could include one or several participants, enabling parents to report on behalf of children <16 years. Participants received weekly e-mails that prompted them to visit the website and record occurrence of 18 listed symptoms or absence of symptoms in the preceding week. Upon affirmation of one or more symptoms, the participant was presented with follow-on questions (e.g. date of symptom onset). The components of the IMS across seasons are presented in Table 1.

**Table 1:** Summary of system components of the IMS and PBS during the influenza seasons 2011–2012 and 2012–2013.

Component	Season 1 (2011–2012)		Season 2 (2012–2013)	
	IMS	PBS	IMS	PBS
Invited sample	–	14,022	2,511	14,558
Invited participants (response proportion %)	–	2,580 (18)	166 (7)	2,236 (15)
Self-recruited participants	2,552	–	2,486	–
Geographical area	Sweden	Stockholm	Sweden	Sweden
Calendar weeks operating	46–20	38–20	47–21	44–20

### The Population-based System (PBS)

Population-based surveillance uses a sample from the general population, defined by geopolitical boundaries which constitutes the denominator, and/or the sampling frame [15]. In the PBS we collected data directly from individuals that had been recruited for the surveillance, based on a random sample of the general population. Descriptions and evaluations of the PBS have been presented previously [4], [5], [16]. Briefly, each year we invited representative samples of the general population, 3 months through 95 years of age to participate in PBS. Participants were instructed to spontaneously report all new episodes of colds and fevers within seven days of symptom onset from September/October through May via a secure website or a telephone based interactive voice response system. When reporting, participants answered an automated, tree-structured symptom questionnaire. Due to non-participation, there is moderate over-representation of elderly people, women, well-educated individuals, people with a high household income, married people, and people living in two-person households [4]. Evaluations have shown that the telephone service seems to be particularly attractive for elderly and low-educated people, but the reporting technology per se does not appear to affect the reporting [4]. A validation study revealed substantial under-reporting, which was remarkably constant over time and across seasons, thus allowing simple adjustments [5].

After confinement to Stockholm County for five years, PBS was extended to all of Sweden in the 2012–2013 season. In season 1, 2,580 out of 14,022 persons sampled from Stockholm’s population participated in PBS. In season 2, 2,236 out of 14,558 people sampled from all over Sweden participated. The components of the PBS across seasons are presented in Table 1. The age and sex distributions of PBS participants during season 1 and 2 are available in Table S2.

### Evaluation Analysis

All analyses were based on data collected between November and May in season 1 and season 2, respectively. Each season lasted 27 weeks and was analysed independently.

All IMS participants who created an account in the system but never submitted a weekly report, participants with missing information on postcode, and participants with a birth date in the future, were excluded from analysis. Further, incomplete reports, reports where symptom onset preceded participation, and reports with a future date of symptom onset were excluded.

#### Definitions

Self-selection into IMS may lead to preferential inclusion of people with symptoms and preferential re-entry of people with symptoms after temporary periods of non-reporting. Therefore, we only included reports preceded by at least one report in the previous three weeks (henceforth referred to as *active* reports) for our incidence calculations. To explore how the definition of active participation affected disease patterns, we defined a *strictly active* report as a report preceded by two consecutive reports in the previous two weeks in a supplementary analysis (henceforth referred to as *strictly active*).

We defined a report of illness in IMS or PBS as ILI if it included sudden onset of symptoms AND at least one of the following systemic symptoms: fever or feverishness, headache, or myalgia, AND at least one of the following respiratory symptoms: cough, sore throat, shortness of breath, or coryza. Coryza was omitted from the case definition in season 2. We calculated the weekly incidence proportions (%) among all IMS participants by dividing the number of *active* ILI reports by the total number of *active* reports in that week. We calculated the weekly incidence proportions among PBS participants by dividing the number of ILI reports by the total number of cohort members in that week. We corrected PBS ILI rates for previously estimated misrepresentation of demographic substrata [4] and under-reporting [5].

To evaluate the performance of IMS for surveillance in close-to-real-time, episodes that started >7 days before the reporting date were excluded from the analysis. Symptoms fitting the case definition in two consecutive weekly reports were considered to represent the same episode of illness and only the first report was included in the analysis.

#### Acceptability by participants

To measure the acceptability [17] in terms of willingness of persons to participate in IMS, we calculated the proportion of participation among the invited participants. We also calculated the weekly response proportion among all enrolled IMS participants by dividing the number of reports during the week in question by the accumulated number of participants enrolled up to that week (for all reports and for *active* reports only, separately for each season). In season 2, we also stratified by self-recruitment or recruitment by invitation. The reports and *active* reports were summarized by the total, mean and median number per participant across each season. Additionally, we calculated the median proportion of complete reports per individually acquired participation time (time from registration week until the season’s last week).

#### Representativeness of the participants

To assess the representativeness [17] of self-recruited and invited IMS participants, we compared participants and the general Swedish population in terms of distributions of age, sex, level of education, and county of residence, using chi-square tests. We analysed invited and self-recruited IMS participants separately. For the invited sample in season 2, we collapsed the 21 counties of residence into three regions (*Götaland*, Southern Sweden; *Svealand,* Central Sweden; and *Norrland*, Northern Sweden) due to small numbers in many counties. We performed a supplementary analysis of representativeness including only participants who had contributed at least one *active* report. We considered p-values <0.05 as significant.

#### Time series of ILI data

We compared ILI occurrence data from IMS (based on reports from all IMS participants) to incidence data from PBS (corrected for estimated demographic misrepresentation [4] and underreporting [5]). To examine the possible presence of systematic differences between the two methods that could be amenable to simple calibration, we compared incidence proportions week by week and across seasons with particular reference to periods with known increased influenza activity. We applied Bland-Altman plots [18] and method comparison techniques [19] to determine if observations from both methods directly agreed, and if not, if they agreed after mathematical transformation of the data. We also studied cross-correlations of the incidence proportions [20]. Further, we studied the cross-correlation of IMS and PBS incidence proportions with ILI data generated by the GP-based sentinel surveillance system (weekly number of ILI cases per 1000 000 listed patients) and laboratory reports (number of laboratory-confirmed influenza cases per week). Before analysis, we smoothed the weekly incidence proportions using a two-week moving average. We plotted each time series and calculated Spearman correlation coefficients (r) on ranked data for different lags (+/−5 weeks) between: IMS and PBS; IMS and laboratory data; PBS and laboratory data; IMS and GP-based sentinel data; and PBS and GP-based sentinel data.

Since PBS was applied only in Stockholm County during season 1, we also restricted IMS data to Stockholm County only. However, due to small numbers in the GP-based sentinel data from Stockholm, these were not included in the season 1 analysis. For season 2, we included all four surveillance systems and made all comparisons at the national level.

In order to examine if the time series comparison would improve after attempts to correct the incidence proportion according to the general Swedish population, we performed a supplementary analysis based on weighted IMS data. We weighted the IMS sample by assigning each participant a weight calculated with the formula [21] **W**_participant_ = **P**_Swedish population_/**P**_IMS participants_ (where W_participant_ = weight of each IMS participant, P_Swedish population_ = proportion of the general population of Sweden in the same age and sex group as the participant and P_IMS participants_ = proportion of the IMS sample in the same age and sex group as the participant).

## Results

### Acceptability

During, season 1 and season 2, respectively 2,552 and 2,486 self-recruited IMS participants submitted at least one report. Of 2,511 randomly selected residents who were invited to IMS, 166 (6.6%) signed up to participate and submitted at least one report.

In season 1, as the number of participants increased, the number of reports per week increased gradually until week 9 of 2012, when it started decreasing (Table 2). The weekly proportion of participants reporting was highest (87%) in the first week but fell almost monotonically to its lowest value (23%) in the last week of the season. The reporting proportion counting only *active* reports increased to 50% after the first three weeks with comparable levels in the following three weeks, but then it gradually fell to its lowest point (21%) in the last week. The median number of total reports and *active* reports per participant was 4 (range 1–27) and 3 (range 0–26). During calendar weeks 8, 9, and 10, coinciding with the season’s influenza peak, the cumulative number of reporting participants increased, and the *active-to-total* report ratio was the greatest. Many participants also joined the system in these weeks. Based on the individually acquired participation time, the median completion proportion of all possible reports and *active* reports were 27% (range: 4–100) and 17% (range: 0–96) respectively.

**Table 2:** The weekly number of reports and reporting proportion among self-recruited and invited IMS participants during the 2011–2012 and 2012–2013 influenza seasons.

	2011–2012			2012–2013					
	Self-recruited participants			Self-recruited participants			Invited participants		
Week	N	Reports (%)	Active* reports (%)	N	Reports (%)	Active* reports (%)	N	Reports (%)	Active* reports (%)
46	304	263 (87)	0 (0)	–	–	–	–	–	–
47	559	469 (84)	180 (32)	237	0 (0)	0 (0)	45	0 (0)	0 (0)
48	750	505 (67)	317 (42)	817	673 (82)	0 (0)	118	100 (85)	0 (0)
49	837	510 (61)	420 (50)	1,000	823 (82)	550 (55)	149	124 (83)	83 (56)
50	873	518 (59)	474 (54)	1,065	803 (75)	721 (68)	152	127 (84)	119 (78)
51	891	420 (47)	397 (45)	1,183	862 (73)	739 (62)	159	129 (81)	119 (75)
52	908	464 (51)	423 (47)	1,287	854 (66)	733 (57)	161	127 (79)	125 (78)
1	984	482 (49)	392 (40)	1,554	1,135 (73)	855 (55)	162	122 (75)	120 (74)
2	1,038	588 (57)	507 (49)	1,807	1,388 (77)	1,102 (61)	164	128 (78)	126 (77)
3	1,151	603 (52)	475 (41)	1,861	1,328 (71)	1,257 (68)	164	130 (79)	129 (79)
4	1,265	713 (56)	583 (46)	1,926	1,333 (69)	1,252 (65)	164	131 (80)	131 (80)
5	1,404	750 (53)	585 (42)	2,015	1,359 (67)	1,251 (62)	164	114 (70)	112 (68)
6	1,446	737 (51)	668 (46)	2,152	1,453 (68)	1,292 (60)	164	138 (84)	135 (82)
7	1,510	723 (48)	641 (42)	2,248	1,456 (65)	1,334 (59)	164	126 (77)	125 (76)
8	2,009	1,203 (60)	665 (33)	2,322	1,459 (63)	1,353 (58)	164	123 (75)	122 (74)
9	2,322	1,267 (55)	914 (39)	2,364	1,432 (61)	1,369 (58)	164	128 (78)	126 (77)
10	2,417	1,095 (45)	970 (40)	2,397	1,403 (59)	1,343 (56)	164	124 (76)	124 (76)
11	2,447	960 (39)	911 (37)	2,421	1,393 (58)	1,332 (55)	164	128 (78)	124 (76)
12	2,473	924 (37)	868 (35)	2,437	850 (35)	825 (34)	164	71 (43)	70 (43)
13	2,496	907 (36)	833 (33)	2,445	1,053 (43)	1,013 (41)	164	108 (66)	108 (66)
14	2,518	669 (27)	623 (25)	2,453	1,305 (53)	1,243 (51)	164	117 (71)	117 (71)
15	2,532	822 (32)	762 (30)	2,462	1,136 (46)	1,072 (44)	164	115 (70)	113 (69)
16	2,538	760 (30)	719 (28)	2,464	1,254 (51)	1,199 (49)	164	126 (77)	125 (76)
17	2,545	795 (31)	731 (29)	2,471	1,161 (47)	1,110 (45)	164	119 (73)	118 (72)
18	2,548	782 (31)	729 (29)	2,476	1,331 (54)	1,259 (51)	165	130 (79)	129 (78)
19	2,549	680 (27)	653 (26)	2,481	1,130 (46)	1,095 (44)	165	113 (68)	113 (68)
20	2,552	585 (23)	542 (21)	2,482	1,152 (46)	1,119 (45)	166	113 (68)	112 (67)
21	–	–	–	2,486	1,114 (45)	1,077 (43)	166	122 (73)	122 (73)
Total	2,552	19,194	15,982	2,486	29,623	27,495	166	3,133	2,947
Median** (range)		4 (1–27)	3 (0–26)		13 (1–26)	11 (0–25)		21 (1–26)	20 (0–25)
Mean**		8	6		12	11		19	18

*Active as defined in the Methods section.

**Number of reports per participant.

In season 2, the number of reports per week also increased gradually in the beginning, when the influx of participants was greatest (Table 2). In contrast to season 1, however, the weekly number of reports remained constant throughout the season, with the exception of a dip in calendar week 12 due to a technical malfunction of the website. Notwithstanding this stability, the weekly reporting proportion among self-recruited IMS participants fell slowly across the season, from its highest (82%) in the first two weeks to 45% in the last week. The reporting proportion counting only *active* reports peaked (68%) in weeks 50 and 3 and was 43% in the last week. The median number of total reports (13, range 1–26) and of *active* reports (11, range 0–25) was higher than in season 1. Based on the individual participation duration, the median completion proportion of all possible reports and *active* reports among self-recruited IMS participants were 64% (range: 4–100) and 57% (range: 0–96) respectively.

In season 2, the median number of reports and *active* reports per registered participant were, respectively, 62% (21 vs. 13, p<0.01) and 82% (20 vs. 11, p<0.01) higher among invited IMS participants than among the self-recruited ones. Disregarding weeks 12 and 13 (affected by the malfunctioning website in week 12), the lowest proportion of participants reporting counting only *active* reports among the invited participants (67% in week 20) was of the same magnitude as the highest proportion among the self-recruited (68% in week 3). Based on the individual participation duration, the median completion proportion of all possible reports and *active* reports among invited IMS participants were 84% (range: 4–100) and 79% (range 0–96) respectively.

### Representativeness

For both seasons and irrespective of how participation was defined, self-recruited IMS participants were more likely to be female, university educated and aged 40–64 than the general population (p<0.01 for each comparison, Table 3). The geographical distribution of participants differed from the Swedish population (p<0.01). For instance, 29% (season 1) and 34% (season 2) of the self-recruited participants resided in Stockholm County compared with only 22% of the Swedish population. In both seasons, only 11% of participants resided in the Swedish county containing the second largest city Gothenburg; this county accommodates 17% of the Swedish population.

**Table 3:** Distribution of socio-demographic characteristics among self-recruited and invited IMS participants during the 2011–2012 and 2012–2013 influenza seasons and the corresponding distribution of the general Swedish population 2011 and 2012.

	2011–2012					2012–2013									
	Self-recruited participants				Sweden 2011	Self-recruited participants				Invited participants				Sweden 2012	
Indicator	N (%)	P*	N (*Active* **)(%)	P*	N (%)	N (%)	P*	N (*Active* **)(%)	P*	N (%)	P*	N (*Active* **)(%)	P*	N (%)	
Age group (yrs)															
0–17	294 (12)	<0.01	199 (11)	<0.01	1,901,291 (20)	76 (3)	<0.01	57 (3)	<0.01	20 (12)	<0.01	19 (12)	<0.01	1,860,527 (19)	
18–39	934 (37)		589 (33)		2,711,405 (29)	875 (35)		698 (33)		60 (36)		52 (34)		2,777,239 (29)	
40–64	1,133 (44)		828 (46)		3,065,375 (32)	1,336 (54)		1,154 (55)		66 (40)		62 (41)		3,087,669 (32)	
65+	184 (7)		160 (9)		1,798,034 (19)	183 (7)		178 (8)		19 (11)		19 (12)		1,793,463 (19)	
Missing	7 (0)		6 (0)		0 (0)	16 (1)		13 (1)		1 (1)		1 (1)		33,477 (0)	
Sex															
Men	898 (35)	<0.01	609 (34)	<0.01	4,723,159 (50)	793 (32)	<0.01	673 (32)	<0.01	76 (46)	0.29	68 (44)	0.18	4,760,835 (50)	
Women	1,654 (65)		1,173 (66)		4,752,946 (50)	1,693 (68)		1,427 (68)		90 (54)		85 (56)		4,785,613 (50)	
Missing	0 (0)		0 (0)		0 (0)	0 (0)		0 (0)		0 (0)		0 (0)		5,927 (0)	
Education (yrs)***															
<9	101 (4)	<0.01	67 (4)	<0.01	1,862,355 (20)	124 (5)	<0.01	98 (5)	<0.01	16 (10)	<0.01	15 (10)	<0.01	1,810,813 (19)	
10­12	420 (16)		264 (15)		3,348,458 (35)	473 (19)		394 (19)		45 (27)		41 (27)		3,378,533 (35)	
13–15	475 (19)		331 (19)		1,000,336 (11)	502 (20)		407 (19)		30 (18)		27 (18)		1,020,584 (11)	
>15	1,221 (48)		896 (50)		1,426,598 (15)	1,322 (53)		1,145 (55)		65 (39)		60 (39)		1,476,228 (15)	
Missing****	335 (13)		224 (13)		1,838,358 (19)	65 (3)		56 (3)		10 (6)		10 (7)		1,866,217 (20)	
Total	2,552 (100)		1,782 (100)		9,476,105 (100)	2,486 (100)		2,100 (100)		166 (100)		153 (100)		9,552,375 (100)	

*Chi square goodness of fit test participants vs. Swedish population.

**Participants who contributed with at least one *active* report. For definition of active reports, see Methods section.

***Among participants 16–95+ year old.

****Including children in age group 0–15 yrs.

The age and sex distributions among invited IMS participants differed less from the Swedish population than did the corresponding distributions among the self-recruited (Table 3). The under-representation of the 0–17 and ≥65 year age groups, though statistically significant, was less marked (p = 0.01). With reservation for the small numbers, the geographical distribution of invited participants was similar to that of the Swedish population (p = 0.75).

### Comparison of Time Series

#### Season 1 (Stockholm County 2011–2012)

**Figure 1:**
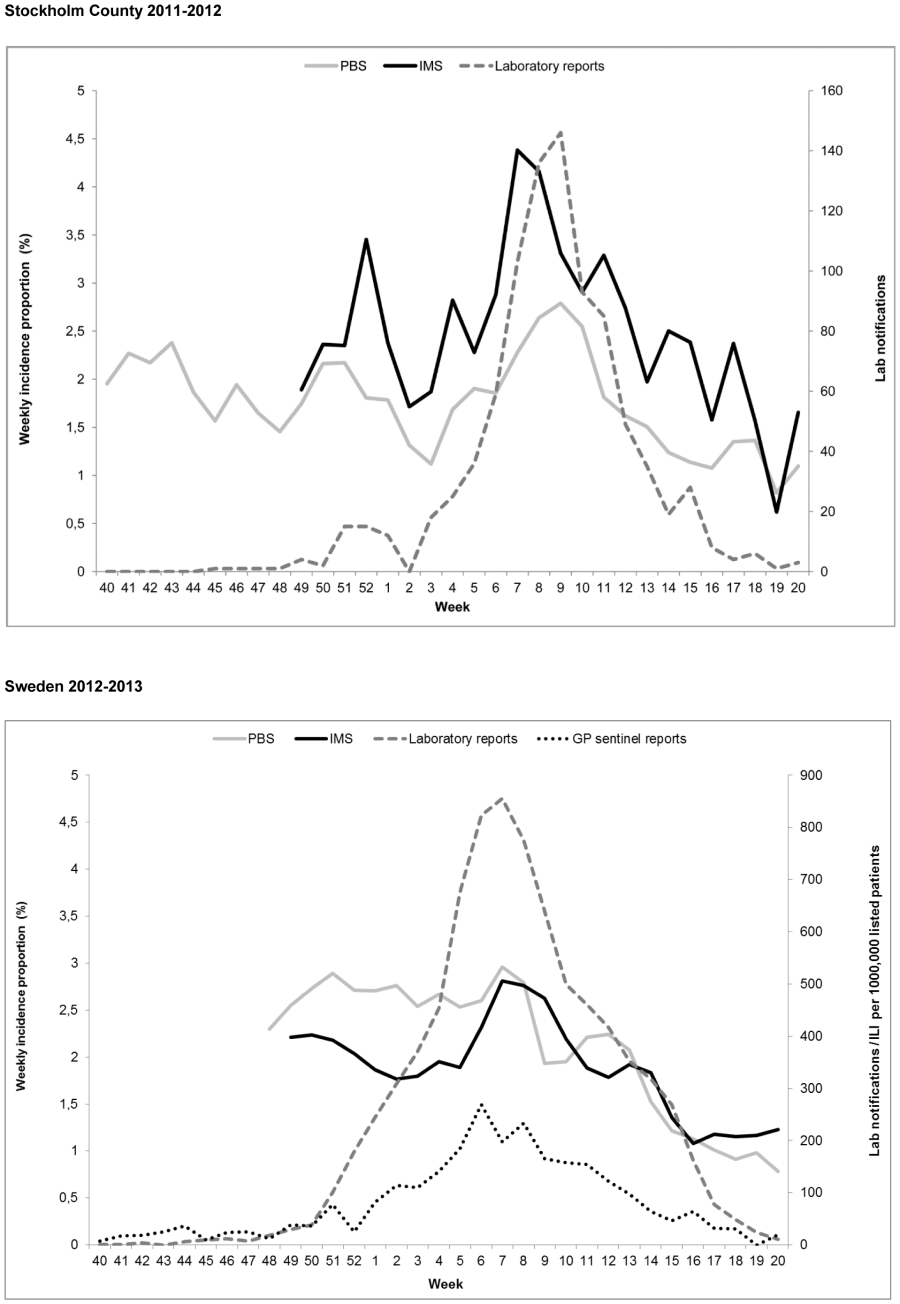
Epidemic curves 2011–2012 and 2012–2013. The upper graph shows the smoothed weekly ILI incidence proportions generated by IMS and PBS (corrected for estimated demographic misrepresentation [4] and underreporting [5]) and number of laboratory confirmed influenza cases, Stockholm 2011–2012. The lower graph shows the smoothed weekly ILI incidence proportions generated by IMS (based on self-recruited and invited participants) and PBS (corrected for estimated demographic misrepresentation [4] and underreporting [5]), number of laboratory confirmed influenza cases, and ILI per 1,000,000 listed patients in GP-sentinel reports, Sweden 2012–2013.

Smoothed weekly ILI incidence proportions ranged between 0.6–4.4% (IMS) and 0.8–2.8% (PBS). IMS reached its peak in week 7, whereas PBS and laboratory reports of influenza diagnoses reached their peaks in week 9 (Figure 1).

IMS correlated with PBS (p<0.05) with the largest coefficient (r = 0.71) when no lag was applied. The correlation was still significant with a shift of PBS data back in time (lead) by up to two weeks (r = 0.56) and with shift of PBS data forward in time (lag) by one week (r = 0.59). IMS correlated with laboratory data (p<0.05) with the highest correlation without a lag (r = 0.77). The correlation was till significant with a shift of laboratory data by two weeks back in time (r = 0.40) and by one week forward in time (r = 0.65). PBS also correlated with laboratory data (p<0.05) with the highest correlation without a lag (r = 0.63). However, correlations were also significant with a shift of laboratory data by one week back in time (r = 0.49) and two weeks forward in time (r = 0.46).

#### Season 2 (Sweden 2012–2013)

The corresponding curves for season 2, pertaining to all of Sweden, also include the weekly number of ILI cases among listed patients reported in the GP-based sentinel system (Figure 1). The smoothed weekly incidence proportions of reported ILI ranged between 1.1–2.8% in IMS and 0.7–3% in PBS.

IMS correlated with PBS (p<0.05) with the maximum coefficient (r = 0.69) when no lag was applied, but the correlation remained significant with a shift of PBS data back in time by up to two weeks (r = 0.54) and forward in time by up to two weeks (r = 0.47). IMS correlated with laboratory data (p<0.05) with the maximum coefficient without a lag (r = 0.56) and with GP sentinel data with a shift of GP sentinel data one week back in time (r = 0.61). Correlations were also significant between two weeks lead (laboratory: r = 0.44, sentinel: r = 0.54) and two weeks lag (laboratory: r = 0.48, sentinel: r = 0.51) of laboratory and sentinel data, respectively. PBS correlated with laboratory and sentinel data (p<0.05) with the maximum coefficient at a four week lag for laboratory data (r = 0.50) and sentinel data (r = 0.55), respectively. Correlations were also significant between one (r = 0.42) and five weeks lag (r = 0.49) of laboratory data and between zero (r = 0.47) and five (r = 0.50) weeks lag of sentinel data.

#### Exploring a stricter definition of active participation

When applying stricter criteria to define a report as *strictly active*, as opposed to the *active* definition used in the main analysis, the IMS weekly incidence proportions were overall lower in both seasons, but the differences were generally small. In season 1, estimates of ILI incidence proportions based on *active* reports were on average 0.6 percentage points (median 0.6 range: −0.8 to 1.8) higher than those derived from *strictly active* reports. In season 2, the differences were generally smaller, on average 0.1 percentage units (median 0.1 range: −0.2 to 0.4), and of similar size across the entire season.

#### Exploring time-series comparison based on weighted IMS-data

The smoothed weekly weighted ILI incidence proportions ranged between 0.9–4.9% in season 1 and 1.3–3.0% in season 2. In both seasons, the weighted incidence proportions were similar to the crude incidence proportions for most of the weeks. However, the weighted data produced peaks in the beginning of both seasons at a similar levels of incidence proportions as the peak that coincided with the influenza peak according to laboratory data. Additionally in season 1, the weighted data produced a peak towards the end of the season. Weighted IMS data correlated weaker to all the other surveillance data sources than the crude IMS data did (data not shown).

#### Difference between weekly estimates

**Figure 2:**
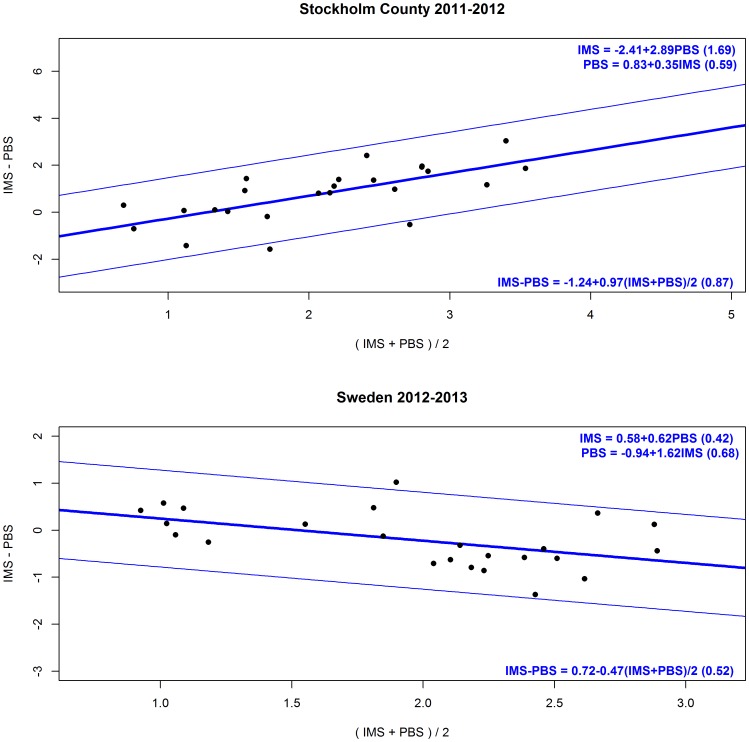
Bland-Altman plots 2011–2012 and 2012–2013. The upper graph shows a Bland-Altman plot of data from the 2011–2012 season and the lower graph shows a Bland-Altman plot of data from the 2012–2013 season. The black dots represents the differences of the weekly incidence proportions between the IMS and PBS (y) by the average of the IMS and PBS weekly incidence proportions (x). The thick blue line represents a simple linear regression model of the differences on the averages, while the thin blue lines represent the respective 95% limits of agreement. The limits of agreement for the difference between the IMS and PBS can be calculated from the equation in the bottom of the graphs, when their average is known. With the equations at the top of the graphs one system’s incidence proportions can be transformed to the other.

In season 1, the weekly estimates of ILI incidence proportions generated by IMS were on average 0.9 percentage units (median 1.0) higher than those derived from the PBS. By and large, the differences were constant across the season, with the greatest (but also the most variable) differences in the weeks when influenza activity was increasing according to laboratory reports. The differences tended to subside towards the end of the season. The Bland-Altman plot suggested that the two systems did not agree, but rather that IMS tended to give higher ILI estimates than PBS (Figure 2). The transformation of IMS to PBS (PBS = 0.83+0.35*IMS) had 95% prediction limits of magnitude ±1.18%; i.e. the true PBS incidence proportion would fall within ±1.18% of the estimate with 95% certainty.

In contrast, in season 2 the mean and median differences were −0.25 and −0.15 percentage points, respectively, indicating that PBS generated higher estimates most of the weeks, but the differences were generally smaller. The greatest differences were seen in the beginning of the season and when influenza activity had passed its peak according to the laboratory reports. A Bland-Altman plot suggested that IMS tended to give higher estimates when the average of IMS and PBS incidence estimates were below 1.5%, and that PBS generated higher estimates for higher means (Figure 2). The 95% prediction limits after transformation of IMS to PBS (PBS = −0.94+1.62*IMS) were of magnitude ±1.36%.

## Discussion

This evaluation of IMS suggested that self-recruitment led to an overrepresentation of women, highly educated and middle-aged persons. The weekly proportion of participants that reported decreased gradually throughout both seasons. Furthermore, only a small proportion of the invited sample participated in IMS. Although the generated ILI-estimates differed from PBS estimates, especially the first season, IMS data correlated significantly with PBS data and with data from the traditional influenza surveillance systems.

Our findings are consistent with previous assessments of Influenzanet that suggested that self-recruited layperson-based influenza surveillance systems could detect changes in ILI incidence in the population to give signals of the start and culmination of influenza epidemics with reasonable accuracy [6], [7], [9]–[11]. The present study adds by providing a comparison with a well-validated population-based surveillance system that generated incidence data in real time, corrected for previously quantified demographic misrepresentation [4] and under-reporting [5].

A recent multi-country analysis of data from seven Influenzanet countries (including Sweden), found that participants with fewer years of education and of younger ages had a lower compliance to complete the weekly report [22], suggesting that further selection bias may be introduced as the season proceeds. This is illustrated in our analysis as the accumulated number of self-recruited IMS participants did not parallel the number of reports and *active* reports submitted each week. Interestingly, the difference between the total number of reports and the total number of *active* reports was generally small, and the total number of reports was fairly stable across the entire season, particularly in season 2. The majority of participants in season 2 responded to more than half of their required reports further implying that the overwhelming number of reports came from faithful participants who reported regularly. Possibly, they reported during limited periods not necessarily starting at the beginning of the season. A notable exception was the period from February through March during season 1, when influenza peaked [12]. At this time, the *active*-to-total report ratio, in particular, fell noticeably. Many participants entered the system, possibly prompted by their own ILI and/or by increased attention to IMS produced by the peak itself.

The requirement of at least one report in the three weeks preceding each new report does not entirely rule out preferential re-entry of sick participants. However, requiring two consecutive reports in the preceding two weeks, practically precluding preferential re-entry of sick participants, resulted in incidence curves with shapes that were trivially different from those based on the more relaxed *active* report definition. This suggests that re-entry may only be a minor problem. Nevertheless, weak selection bias in IMS during the influenza peak might have amplified and improved the signal, thus making the peak more distinct in IMS than in PBS.

The reporting activity level among IMS participants improved in season 2. This may be due to inclusion of motivated participants from the previous season and a concentration of marketing efforts to the beginning of the season rather than continuously. The higher reporting frequency in the invited sample indicates that this group was even more motivated to report regularly. Although a seven percent response proportion among the invited residents likely enriched particularly motivated participants, the detailed information in the postal invitation may also have contributed to the regular reporting. Furthermore, the step from receiving the paper invitation to registering online may have demanded more motivation to participate than reading about IMS online, where registration is only “a mouse click away”.

The underrepresentation of the youngest age groups among self-recruited IMS participants may be due to modest emphasis on the possibility for legal guardians to act as proxy participants for their children. In the oldest age groups, limited Internet availability and computer literacy may have prevented participation [23]. Interestingly, despite the poor participation rate, the randomly selected, invited population sample was more representative with regard to age, sex and education.

Notwithstanding the misrepresentation of self-recruited IMS participants, the overall epidemic curves were similar to those generated by PBS. This suggests that neither the measured socio-demographic factors nor unmeasured determinants of participation were strongly associated with the risk of ILI. While IMS may detect the start and peak of influenza epidemics, the continuous monitoring of absolute incidence rates in various substrata of the population may be better accomplished with PBS. Age group-specific data is of particular interest because immunity and the predisposition to complications differ across ages [24]. Since the representation of specific age groups in self-recruited IMS is variable and poor in the elderly, the validity of incidence data in this group is uncertain. The pattern of misrepresentation of participants was similar in PBS [4], but the deviations were smaller compared with self-recruited IMS. Phone reporting offered by PBS may explain better representation of older age groups [4]. When weighting the IMS sample according to the age and sex distribution of the Swedish population, the epidemic curves deviated more from the PBS. The weighted estimates may give a less biased cross-sectional estimate, but interpretation of time series stays as complicated as for crude estimates due to the weekly variations in stratum specific reporting activity. However, it is reassuring that even misrepresented self-selected populations are capable of describing an epidemic of influenza-like illness with a reasonable accuracy.

The differences between incidence proportions generated by IMS and PBS varied across and between the seasons. Based on the Bland-Altman plots and method comparison techniques, the prediction limits in the transformation of the estimates of one system to the other were of a magnitude that we consider unacceptable for the purpose of surveillance. Notably, the incidence according to PBS was unprecedentedly high from November until the peak in March of season 2 and did not coincide with the laboratory confirmed influenza data. The reasons for this deviation of PBS data compared to previous seasons remain speculative but may relate to the epidemiology of respiratory infections. First, the 2012–2013 influenza season was unusually long, with three circulating strains that affected all age groups [25]. The PBS may have picked up a higher baseline activity of ILI that was missed by IMS because of an under representation of older and younger age groups. Second, the higher baseline activity of ILI in PBS in the first part of season 2 coincided with the start of the respiratory syncytial virus (RSV) season [26], possibly resulting in influenza-like symptoms, mainly among children. Lastly, after having been confined to Stockholm County for five seasons, a PBS surveillance cohort of the same size as the previous cohorts in Stockholm was drawn from the whole country (0.12% of the Stockholm population vs 0.02% of the Swedish population). Local and regional variations in influenza surveillance and epidemiology may have affected comparability.

### Limitations

The elements assessed in this evaluation provided insights about the functionality of IMS and illustrated differences between the two community-based surveillance systems. However, structured evaluation of other aspects, such as timeliness, flexibility, stability and resources needed, may provide further understanding about the usefulness of IMS [17]. Moreover, evaluation of only two seasons that provided somewhat deviating findings, possibly due to differences in the geographical distribution of the sample and during some periods the small sample size, makes generalisation of the results difficult.

### Conclusion

In conclusion, the self-recruited IMS participants reflected the demography of the Swedish population poorly. Yet IMS offered a reasonable representation of the temporal ILI pattern in the community overall during the 2011–2012 and 2012–2013 influenza seasons and could be a simple tool to collect community-based ILI data. However, invited IMS participants represented the target population better than the self-recruited and completed a larger proportion of reports. Therefore, personal invitations to a random sample of the population may improve the quality and usability of IMS surveillance data.

## Supporting Information

Table S1 Distribution of socio-demographic indicators among invited residents and invited IMS participants, Sweden 2012–2013.
(PDF)

Table S2 Distribution of age and sex among PBS participants during the 2011–2012 and 2012–2013 influenza seasons.
(PDF)
