# Supplementary material for: Evaluation of an Internet-Based Monitoring System for Influenza-Like Illness in Sweden
Source: PLoS One. 2014 May 13;9(5):e96740. doi: 10.1371/journal.pone.0096740 (PMC4019478; doi:10.1371/journal.pone.0096740)
Supplement: Table S1 — Distribution of socio-demographic indicators among invited residents and invited IMS participants, Sweden 2012–2013. (PDF) [file pone.0096740.s001.pdf]

Table S1: Distribution of socio-demographic characteristics among invited residents and invited IMS participants, Sweden 2012- 2013.

Indicator	Sample N (%)	Invited IMS-participants N (%)
Age group (yrs)		
0-19	608 (24)	21 (13)
20-39	987 (39)	59 (36)
40-64	689 (27)	66 (40)
65+	227 (9)	19 (11)
Missing	0 (0)	1 (1)
Sex		
Men	1,252 (50)	76 (46)
Women	1,259 (50)	90 (54)
Education (yrs)*		
<9	440 (18)	16 (10)
10-12	879 (35)	45 (27)
13-15	262 (10)	30 (18)
>15	390 (16)	65 (39)
Missing**	540 (22)	10 (6)
Total	2,511	166

*Among residents and participants 16-95 year old

** Including children in age group 0-15 yrs
