# Supplementary material for: Evaluation of an Internet-Based Monitoring System for Influenza-Like Illness in Sweden
Source: PLoS One. 2014 May 13;9(5):e96740. doi: 10.1371/journal.pone.0096740 (PMC4019478; doi:10.1371/journal.pone.0096740)
Supplement: Table S2 — Distribution of age and sex among PBS participants during the 2011–2012 and 2012–2013 influenza seasons. (PDF) [file pone.0096740.s002.pdf]

Table S2: Distribution of age and sex among PBS participants during the 2011-2012 and 2012-2013 influenza seasons.

	PBS- participants	
**Indicator**	**Stockholm County 2011-2012 N (%)**	**Sweden 2012-213 N (%)**
Age group (yrs)		
0-17	696 (27)	393 (18)
18-39	764 (30)	556 (25)
40-64	738 (29)	998 ((45)
65+	382 (15)	289 (13)
Sex		
Men	1,054 (41)	976 (44)
Women	1,526 (59)	1,260 (56)
Total	2,580 (100)	2,236 (100)
